# Potential Regulatory Roles of GRK2 in Endothelial Cell Activity and Pathological Angiogenesis

**DOI:** 10.3389/fimmu.2021.698424

**Published:** 2021-07-15

**Authors:** Jiajie Kuai, Chenchen Han, Wei Wei

**Affiliations:** Institute of Clinical Pharmacology, Key Laboratory of Anti-Inflammatory and Immune Medicine (Anhui Medical University), Ministry of Education, Anhui Collaborative Innovation Center of Anti-inflammatory and Immune Medicine, Anhui Medical University, Hefei, China

**Keywords:** GRK2, GPCRs, endothelial cells, activity, angiogenesis

## Abstract

G protein-coupled receptor (GPCR) kinase 2 (GRK2) is an integrative node in many signaling network cascades. Emerging evidence indicates that GRK2 can interact with a large number of GPCRs and non-GPCR substrates in both kinase-dependent and -independent modes. Some of these pathways are associated with endothelial cell (EC) activity. The active state of ECs is a pivotal factor in angiogenesis. The occurrence and development of some inflammation-related diseases are accompanied by pathological angiogenesis, but there remains a lack of effective targeted treatments. Alterations in the expression and/or localization of GRK2 have been identified in several types of diseases and have been demonstrated to regulate the angiogenesis process in these diseases. GRK2 as a target may be a promising candidate for anti-angiogenesis therapy.

## Introduction

G protein-coupled receptor (GPCR) kinases (GRKs) can specifically recognize and phosphorylate agonist-activated GPCRs that follow β-arrestin binding, leading to the uncoupling of heterotrimeric G proteins and receptor desensitization (1). Both GRKs and β-arrestin can interact with a variety of cellular proteins involved in signal transduction, thus promoting signal propagation upon GPCR activation. In addition, GRKs are signaling mediators that are independent of both G protein- and β-arrestin-mediated pathways by phosphorylating and/or interacting with other non-GPCR proteins, including receptor tyrosine kinases (RTKs) and a large number of cytosolic or nuclear signaling components of pathways related to multifarious physiological and physiopathological processes (2, 3).

So far, seven GRKs (GRK1-GRK7) have been found in mammals and all GRK isoforms share similar domains (4). However, these isoforms show differential expression patterns and functions (5). GRK2, one of the most studied GRKs, is expressed ubiquitously throughout the body and is emerging as a key node in multiple signal transduction pathways, displaying a very complex interactome. GRK2 knockout mice are embryonic lethal, while knockout mice for other GRKs are born and grow normally (6). GRK2 can affect the behavior of a variety of cells, including endothelial cells (ECs), through diverse pathways.

Angiogenesis, which is different from vasculogenesis, refers to the formation of new blood vessels from pre-existing blood vessel (7). In vertebrates, angiogenesis is common in various complex physiological and pathological conditions. This process involves a number of signaling cascades that can induce cell activation, and EC activation and migration are essential components (8–10). Excessive angiogenesis is often a key factor in the occurrence and progression of inflammation-related diseases, such as cancer and rheumatoid arthritis (RA). Thus, anti-angiogenic therapy has become a promising approach for these diseases in the last 20 years (11, 12). However, there are still unresolved questions that need further research in this field including its potential targets and predictive biomarkers, use in different types or different stages of disease and potential mechanisms of drug resistance (13, 14).

**Figure 1:**
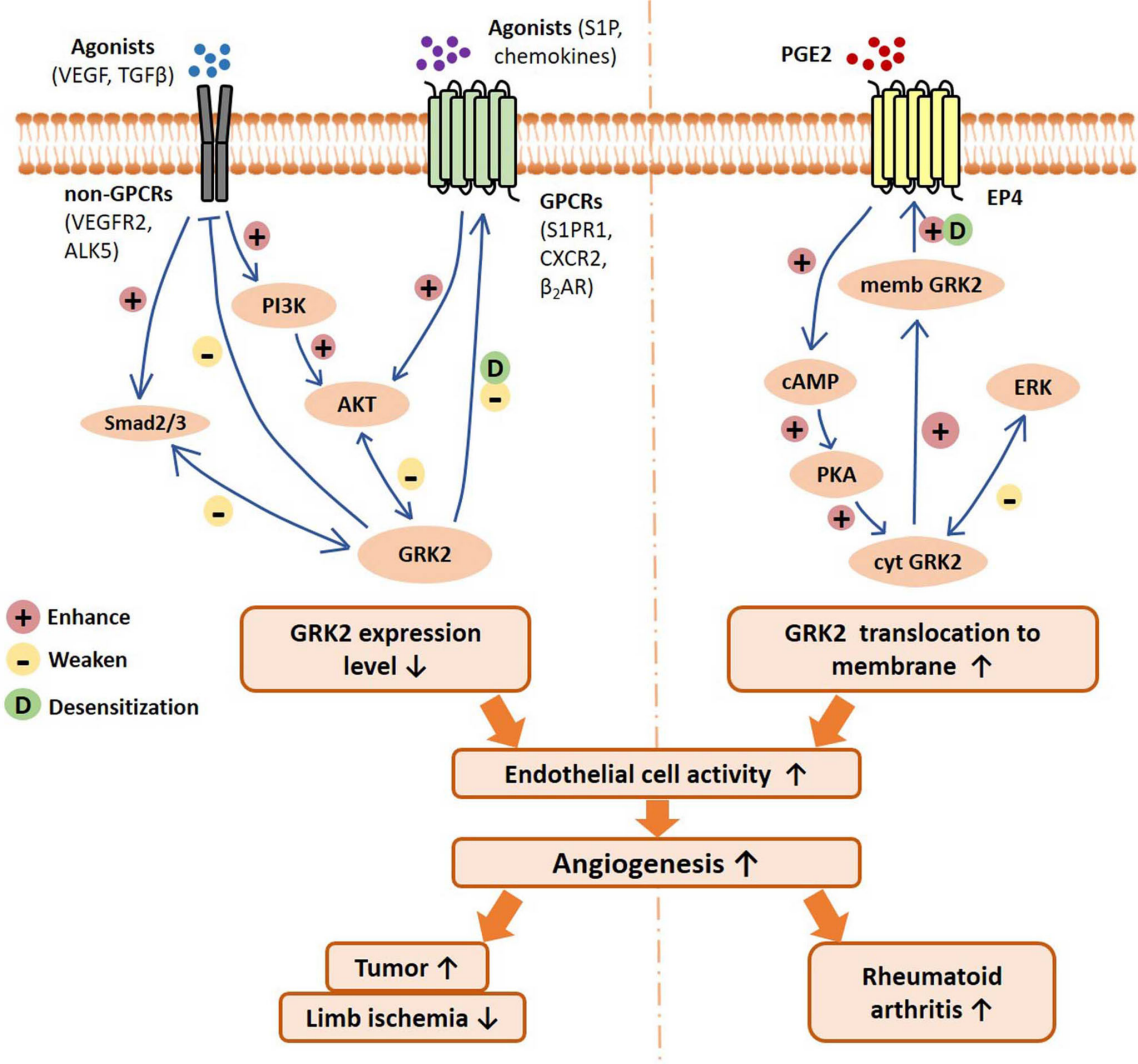
Schematic of GRK2 signaling pathways regulating endothelial cell activity, angiogenesis, and related disease progression. On the one hand, GPCRs and non-GPCRs binding with agonists can activate Smad2/3 signal and PI3K/AKT signaling pathway, resulting in downregulation of GRK2 expression level. Reduced GRK2 expression level reduces GPCR desensitization and the inhibition of non-GPCRs. On the other hand, PGE2 activates the EP4/AC/cAMP/PKA pathway, which mediates GRK2 translocation to the cell membrane, resulting in the reduction of ERK inhibition. In different disease conditions, the decreased GRK2 expression or the increased GRK2 translocation may improve the activity of endothelial cells, and thus promote angiogenesis and disease progression. VEGF, vascular endothelial growth factor; TGFβ, transforming growth factor β; VEGFR2, vascular endothelial growth factor receptor 2; ALK5, TGF-β type I receptor ALK5 (activin receptor-like kinase 5); S1P, sphingosine 1 phosphate; S1PR1, sphingosine 1 phosphate receptor 1; β_2_AR, β_2_-adrenergic receptor; CXCR2, chemokine (C-X-C motif) receptor 2; PGE2, prostaglandin E2; EP4, prostaglandin E2 receptor 4; PI3K, phosphoinositide 3-kinase; AKT, protein kinase B; cAMP, cyclic adenosine monophosphate; PKA, Protein Kinase A; ERK, extracellular regulated protein kinases; memb, membrane; cyt, cytoplasm.

In this review, we discuss the recently reported regulation modes of GRK2 in EC activity, with emphasis on the underlying contribution of this kinase in pathological angiogenesis and relevant disease progression (**Figure 1**). These findings suggest that GRK2 may be a potential target for anti-angiogenic therapy.

## Canonical Roles Of GRK2

GRK2, a ubiquitous member of the GRK family, plays a fundamental role in GRK family proteins (15). The GRK family is a subfamily of AGC (protein kinase A (PKA)/G/C-like) kinases originally defined as inhibitors of GPCR signaling which depend on G proteins, and appear to play a comprehensive regulatory role in signal transduction cascades (16). Upon G proteins binding, GPCRs which constitute the largest family of membrane receptors and are involved in a wide variety of physiological or physiopathological regulation, activate downstream signaling pathways through their coupled Gα subunits. Meanwhile, G proteins activate downstream targets by converting GDP to GTP in response to GPCR conformational changes. GRKs, together with the cytosolic protein β-arrestin, can participate in the regulatory processes of GPCRs by coupling to heterotrimeric G proteins. In particular, ligand-bound GPCRs are specifically phosphorylated by GRKs, and this process leads to the recruitment to phosphorylated receptors of β-arrestin and the inhibition of further G protein activation by causing uncoupling from G proteins, a procedure known as GPCR desensitization.

In addition to the inhibitory role of GRKs/β-arrestin in GPCR signaling, β-arrestin acts as a scaffold protein for several endocytosis adaptors and signaling mediators (2, 17). β-arrestin-bound GPCRs are targeted for endocytosis, which leads to dephosphorylation, re-sensitization and eventual receptor return to the plasma membrane, thus triggering receptor internalization, recycling and the modulation of additional signaling cascades by GPCRs (18). In other words, GPCR desensitization regulated by GRKs can induce GPCRs to participate in G-protein independent signaling cascades that contribute to signal propagation at defined cellular locations upon GPCR activation. However, the molecular mechanism of this event has not yet been fully elucidated. GRKs are believed to be important in β-arrestin-biased signaling, because GRKs are able to promote high-affinity binding of β-arrestin to GPCRs. There are also studies that suggest that different phosphorylation patterns may directly affect β-arrestin–dependent functions, but this still needs to be further explored (19). Therefore, GPCR activation may promote either G-coupled proteins, β-arrestin signaling, or both. GRK-mediated β-arrestin recruitment is critical for triggering the regulation of multiple intracellular signaling cascades by GPCRs, a process that controls the balance between the G protein- and β-arrestin-dependent GPCR signaling cascades.

As an important bridge of intra- and extracellular signal transduction, GPCR signals participate in angiogenesis in pathological conditions, such as ischemic and inflammatory diseases. For instance, a variety of GPCRs and their ligands are involved in tumor angiogenesis, such as angiotensin (Ang) II type I receptor/AngII in breast cancer, sphingosine-1-phosphate receptor 1 (S1PR1)/sphingosine-1-phosphate (S1P) in lymphangiogenesis, CXCR4/CXCL12 in prostate cancer, and CXCR7/CXCL12 in human hepatocellular carcinoma cells (20). However, whether GRK2 participates in these GPCR signaling pathways and angiogenesis remains to be verified.

## GRK2 Has Multiple Physiological Regulatory Functions By Binding To Non-Gpcr Substrates

The role of GRKs in signaling transduction is not limited to the promotion of β-arrestin binding to activated GPCRs. In addition to the canonical roles mentioned above, GRK2, as a common GRK isoform, can initiate noncanonical signaling pathways and participate in the regulation of cell behaviors (cell proliferation, cell differentiation, cell migration, and cell cycle) and related physiological and physiopathological processes by phosphorylating and/or interacting with non-GPCR proteins (2, 21–23). Non-GPCR proteins include single-transmembrane receptors, cytosolic proteins, and nuclear proteins.

Although GRKs display kinase activity, GRKs can also interact with intracellular proteins and modulate downstream signaling pathways in a kinase activity-dependent and -independent manner. In phosphorylation-dependent processes, GRK2 can regulate signaling mediated by other membrane receptor families, except for GPCRs, including RTKs for epidermal growth factor receptor (EGFR) (24) or platelet-derived growth factor receptor (PDGFR) (25). Early evidence of GRK2 as an RTK signaling modulator was based on the observation that the activation of EGFR ligand promotes the translocation of GRK2 to the plasma membrane, thus initiating the internalization of EGFR. GRK2 overexpression can also regulate the signal transduction ability of EGFR in promoting the activation of extracellular regulated protein kinase (ERK)/MAPK *via* phosphorylation (26). The catalytic activity of PDGFR is dependent on the inhibitory activity of GRK2 (27). Moreover, GRK2 also phosphorylates a large number of non-GPCRs: non-plasma membrane receptor substrates, such as p38 MAPK, AKT, histone deacetylase 6 (HDAC6), and insulin receptor substrate (IRS)-1; transcriptional modulators, such as Smad2/3; the calcium-binding protein, such as downstream regulatory element antagonist modulator (DREAM); and cytoskeletal proteins, such as tubulin or ezrin (4, 28).

GRK2 may also contribute to the modulation of cellular responses in a phosphorylation-independent manner as a result of its ability to interact with a plethora of proteins involved in signaling and trafficking, including G protein subunits, such as Gαq and Gβγ; intracellular proteins, such as phosphoinositide 3 kinase (PI3K), MEK, RalA GTPase, GRK interacting protein-1 (GIT-1) and adenomatous polyposis coli (APC) protein; or key players in stress response and survival as murine double minute 2 (Mdm2) (3, 29). These proteins directly interact with GRK2 without phosphorylation and cause various signal cascades and physiological effects. Specifically, the interaction of GRK2 with PI3Kgamma may facilitate PI3K recruitment to the membrane, thus contributing to receptor endocytosis and desensitization (30). The interaction of GRK2/MEK may be important for modulating the chemokine induction of MAPK activation (31). The interaction between GRK2 and GIT-1 may be important for the modulation of cell migration in epithelial cells. These complex GRK2 interactions prove that GRK2 lies at the crossroads of complex signaling pathways and regulation of cellular behavior.

## GRK2 Attends The Modulation Of Endothelial Cell Activity

Recent evidence suggests that GRK2 plays an important role in the activity of various cells by interacting with multiple proteins such as p38 MAPK, MEK, AKT, ezrin, PI3Kgamma, GIT-1 and type 1 insulin-like growth factor receptor (IGF1R) (21, 32). GRK2 phosphorylates manifold chemokine receptors such as CCR5, CCR2b, CXCR4, and CXCR2, as well as chemotactic receptors for substance P, S1P, and formyl-peptide, which is in charge of leukocyte trafficking to the inflammatory foci, T-cell egression from lymphoid organs, and leukocyte activation and proliferation (33). In addition, in epithelial cell lines/fibroblasts, integrins promote sphingosine kinase stimulation leading to paracrine/autocrine activation of S1P receptors, which recruit GRK2 to the plasma membrane and interact with GIT-1. This process can enhance tyrosine phosphorylation of GRK2 and decrease upon phosphorylation by ERK at S670, which activates the Rac/PAK/MEK/ERK cascade and promotes cell migration (34).

GRK2 can also repress the serum-, insulin-like growth factor 1 (IGF1)-, angiotensin (Ang)II-, tumor necrosis factor (TNF) α- or PDGF-induced proliferation and migration of thyroid cancer cell lines (35), human hepatocellular carcinoma cells (32, 36) and smooth muscle cells (27, 28), respectively. For instance, in human hepatocellular carcinoma cell lines (HCCLM3 and HepG2), overexpression of GRK2 inhibits IGF1R signaling activation, the activation of PI3K/AKT and MEK/ERK pathways, and the expression of early growth response protein 1 (EGR1), which results in the suppression of proliferation and migration of cells. However, most of these mechanisms require further exploration.

Regulation of EC behaviors is similar to that of other cells, but possesses a specific set of receptors and ligands to orchestrate these distinct aspects of this process including proliferation, migration, invasion, and permeability (37, 38). In sinusoidal ECs, increased expression of GRK2 interacts with many molecules, which inhibit NO production and further induce portal hypertension (39). There is an increasing number of studies showing that signaling pathways related to GRK2 are involved in common vascular EC activity, especially proliferation and migration, in both kinase-dependent and -independent functions as summarized in the following sections. In this regard, we discussed the potential influence of GRK2 endothelial expression level or localization on cell activity.

### Changes in GRK2 Expression Level Impact Endothelial Cell Activity

The AKT pathway is implicated in vascular endothelial growth factor (VEGF)-A-dependent EC function, including cell survival, proliferation, and generation of nitric oxide, *via* the VEGF receptor-2/PI3K/AKT-PKB axis (40, 41). Interestingly, the expression level of GRK2 in ECs may also be a crucial factor in regulating cell activity through its inhibitory effect on AKT-related signaling pathways upon distinct stimuli. Ex vivo data in murine lung ECs (MLECs) show that GRK2 downregulation enhances signaling to both AKT and ERK cascades under the stimulation of VEGF and S1P, leading to increased EC migration activity (42). Another study that supports the above result showed that downregulation of GRK2 with shRNAs in human umbilical vein ECs (HUVECs) was sufficient to increase the phosphorylated AKT level (43). Furthermore, overexpression of GRK2 increased the amount of GRK2-immunoprecipiated AKT and reduced the levels of activated AKT and CXCR2 in HUVECs. In addition, EC function can be inhibited by GRK2/AKT-dependent β_2_-adrenergic receptor (β_2_AR) dysfunctional signaling (44). In bovine aortic ECs (BAECs), GRK2 overexpression induced a marked increase in p-β_2_AR levels and its subsequent desensitization, and further induced impairment of both cell migration and proliferation. At the molecular level, the deleterious effects of GRK2 on EC function were suggested by the levels of receptor phosphorylation and reduced AKT and eNOS activation.

Thus, these observations implied that the GRK2/AKT interaction mediates EC migration and other activities *in vivo*. The GRK2 expression level may be a negatively regulating factor of EC activity according to the above study results. However, the effect of GRK2 on cell migration is dependent on the cell type, and involves its dynamic interaction with a variety of cellular proteins, leading to differential networks of interaction of GRK2 with cell migration-related signalosomes. Whether these mechanisms apply to other cell types or conditions requires further investigation.

Besides the AKT-related axis, the impact of the GRK2 expression level on EC function is involved in other cytokines and pathways. Knockdown of GRK2 in MLEC results in aberrant endothelial secretion of platelet-derived growth factor (PDGF)-BB and chemokine (C-X-C motif) ligand 12 (CXCL12) (42). PDGF-BB can bind to PDGFR-β on pericytes and through the stimulation of endothelial production of the pericyte chemoattractant CXCL12, which is responsible for the recruitment of pericytes. This study also showed that macrophage migration was further promoted in the conditioned medium of MLECs isolated from Tie2Cre-*Grk2* ^fl/fl^ mice compared with that in WT MLECs, likely related to the increased basal secretion of CXCL12 and other macrophage regulatory and chemoattractant factors such as granulocyte-macrophage colony stimulating factor (GM-CSF) and Factor-III. In addition, GRK2 levels in ECs can modulate TGF-β signaling by controlling ALK1 and ALK5 receptors. GRK2 downregulation restored the ALK5/p-Smad2/3 signaling pathway and inhibited the ALK1/p-smad1/4 signaling pathway. This effect contributes to the imbalance in TGF-β signaling toward the ALK5 route rather than the ALK1 route. All these results mentioned above prove that GRK2 expression level is an important modulator of EC activity, as GRK2 has crosstalk with complex up- and downstream interactions in this process.

### Changes in GRK2 Localization Impact Endothelial Cell Activity

In addition to the expression level, the localization in the membrane (memb) or cytoplasm (cyt) of GRK2 is another critical factor that determines which proteins at a particular location GKR2 interacts with, and eventually influences GPCR signaling and desensitization (15). HUVEC proliferation, migration, and tube formation *in vitro* can be increased by prostaglandin E2 (PGE2)-bound PGE2 receptor 4 (EP4), which promotes GRK2 translocation to the cell membrane (45). In this process, constitutive phosphorylation by protein kinase A (PKA) leads GRK2 to associate with the EP4 receptor and maintain persistent EP4 receptor desensitization. At the molecular level, inhibition of EP4, cyclic adenosine monophosphate (cAMP), PKA or GRK2 significantly blocks the PGE2-dependent increase in the expression of ERK1/2, memb GRK2 and cyt EP4; upregulates the expression of memb EP4 and cyt GRK2; but does not affect GRK2 mRNA expression, a process that inhibits the association of GRK2 and EP4 and increases the association of cyt GRK2 and ERK1/2. These results suggest that PGE2-induced EC activation may occur through the EP4/AC/cAMP/PKA pathway, which mediates GRK2 translocation to the cell membrane, not the GRK2 mRNA expression level, and the reduction of the inhibitory effect of GRK2 on ERK1/2. ERK1/2 can phosphorylate GRK2 on Ser670, inhibiting kinase translocation and catalytic activity towards receptor membrane substrates (34), which may help to maintain the above process. Besides the EP4/AC/cAMP/PKA axis, CXCL12 can also improve HUVEC function by increasing GRK2 translocation, preventing the inhibitory effect of GRK2 on ERK1/2 in the cytoplasm, and stimulating ERK1/2 phosphorylation (46).

Overall, these studies suggest that changes in GRK2 expression and/or localization taking place in ECs may alter GPCRs- or non-GPCRs-involved signaling through a variety of mechanisms, eventually activating or inactivating ECs. EC activation, especially migration, is an essential component of angiogenesis (10, 47). EC migration is regulated directionally by chemotactic, haptotactic, and mechanotactic stimuli and further involves degradation of the extracellular matrix to drive the progression of migrating cells. GRK2 downregulation in ECs causes defective tube formation on Matrigel in mice (42). Therefore, we considered whether GRK2 has an effect on angiogenesis under pathological conditions and further affects disease progression.

## GRK2 Mediates The Regulation Of Angiogenesis In Pathological Conditions

Angiogenesis is a precisely regulated biological event that generates new blood vessels from existing vasculature (8). Under physiological conditions, this process occurs during embryonic development (48), pregnancy, and through the ovarian cycle, but angiogenesis can also be reactivated in a variety of pathological conditions, including cancer (49–51), RA (52–54), ischemia (55, 56) and wound healing (57). The expression and function of GRK2 is tightly modulated, and its level and functionality are altered in several pathological situations (58–60). These changes contribute to EC activation and further angiogenesis in some pathologies, which has been preliminarily proven (**Table 1**).

**Table 1:** Correlation between GRK2 expression and translocation to pathological angiogenesis.

Diseases	Samples sources	GRK2 expression	GRK2 translocation	Angiogenesis process	Related mechanisms
Breast cancer	Patient (42)	Down	ND	Promoted	–
	Cell line (61)	Up	ND	–	HDAC6/Pin1 axis
					AKT/ERK cascades
Melanoma	Mouse (42)	Down	ND	Promoted	Macrophage infiltration
Kaposi’s sarcoma	Mouse (62)	Down	ND	Promoted	Increased expression of essential angiogenesis-related genes
Rheumatoid arthritis	Patient (45)	Up	ND	Promoted	–
	Rat (45)	Up	To cell membrane	Promoted	EP4/AC/cAMP/PKA-mediated
					ERK1/2 activation
Limb ischemia	Rat (44)	Up	ND	Inhibited	βAR-desensitization/down-regulation

ND, not determined.

### GRK2 in Tumor Angiogenesis

Tumor angiogenesis is a hallmark of cancer and plays an essential role in tumor initiation, progression, and metastasis (63, 64). Microvascular density, architecture, and maturity are important factors affecting tumorigenesis and progression. Abnormal vascularization, characterized by hyperplasia, tortuosity, and leakage, has been suggested to lead to excessive hypoxia, which, in turn, can drive tumor cells to acquire stronger growth and invasive capabilities (65, 66). According to the analysis of clinical samples and *in vivo* experiments in mice, GRK2 plays a key role in regulating angiogenesis in a variety of tumors.

In different human breast cancer samples, deficiency of GRK2 staining is predominantly associated with intratumoral vessels, as approximately 60% of vessels exhibit only low or no signals for GRK2, while vessels in the normal tissue surrounding the tumor core have a higher expression of GRK2 protein (42). This indicates that GRK2 expression is suppressed during tumoral, but not normal, angiogenesis, suggesting that GRK2 downregulation could serve as a novel marker for pathological vasculature. Data analysis in the above studies were from different breast carcinoma tumor samples; however, the classification of these breast carcinoma samples was not described in detail. In view of GRK2 upregulation in luminal breast cancer cell lines, in spontaneous tumors in mice, and in some patients with invasive ductal carcinoma (61), confirming the expression levels of GRK2 in perivascular cells and ECs will contribute to clarifying the mechanism by which GRK2 regulates angiogenesis in different types of breast cancer.

In the B16F10 melanoma model, accelerated tumor progression occurs upon GRK2 downregulation, along with immature tumor vessel architecture, increased vessel perimeter, and tortuosity compared with that in wild-type (WT) mice (42). Furthermore, compared with WT animals, tumor vessels growing in endothelial GRK2 knockdown mice were less covered by pericytes, and the microenvironment of these tumors also exhibited a marked increase in hypoxia, adrenomedullin, and higher infiltration of macrophages. GRK2 is involved in T cell infiltration (67), while the results of previous studies suggest that GRK2 may be involved in macrophage infiltration in the tumor microenvironment. Tumor-associated macrophages are divided into two types (M1 and M2 macrophages), which play distinct roles in tumor angiogenesis (68–70). Thus, further studies are needed to elucidate the potential role of GRK2 in tumor angiogenesis by regulating macrophage infiltration.

Kaposi’s sarcoma (KS), the most common tumor type in patients with acquired immune deficiency syndrome, is a highly disseminated angiogenic tumor of ECs linked to infection by KS-associated herpesvirus (KSHV) (71). KS is histologically characterized by an excessively dense and abnormal morphology of blood vessels and vast inflammatory infiltration (72). In a KS mouse model, knockdown of GRK2 not only markedly enhanced KSHV-induced angiogenesis, but also increased the transcriptional expression levels of essential angiogenesis-related genes (43). In contrast, overexpression of GRK2 in BC3 cells impaired the angiogenic capability of KSHV in an *in vivo* Matrigel plug assay (62). In general, studies relating to these three types of tumors suggest that the downregulation of GRK2 is closely related to abnormal angiogenesis in the tumor microenvironment.

### GRK2 in Pannus of Rheumatoid Arthritis

Pannus is the key event and characteristic pathological feature of RA causing joint destruction, and angiogenesis is a critical factor in the development of pannus, and is considered to facilitate hypoxia and extravasation of inflammatory leukocytes, responsible for maintaining the inflammatory condition (53, 73). Based on *in vivo* trials in collagen-induced arthritis (CIA) rats, EP4/AC/cAMP/PKA-mediated GRK2 translocation to the cell membrane and the inhibition of GRK2 by ERK1/2 has been shown to be involved in the regulation of PGE2-dependent proangiogenic processes (45). Moreover, compared with normal synovial tissues (STs), abundant GRK2 protein expression levels, vascular branches, and pannus formation were increased in the STs of patients with RA and in CIA rats. Accordingly, GRK2 upregulation and localization towards the cell membrane may both play a crucial role in the mediation of pathological angiogenesis in RA. Although a suppression of GRK2 protein expression (~55%) and kinase activity was found in peripheral blood mononuclear cells (PBMCs) in patients with RA compared with healthy subjects (74), the translocation of GRK2 in PBMCs and the expression pattern of GRK2 in the pathological areas of joints are of great significance to elucidate the mechanism by which GRK2 regulates angiogenesis and needs further study.

### GRK2 in Limb Ischemia

Pathological ischemia leads to increased sympathetic catecholamine levels, which can further cause β_2_AR signaling dysfunction in ECs, resulting in an insufficient angiogenesis response and loss of vascular tissue integrity and/or function (75, 76). In a rat hind limb ischemia model, ischemia induces the upregulation of GRK2 protein levels in skeletal muscle. This event appears to be critical in the process of revascularization of the ischemic hind limbs as it is closely associated with βAR-desensitization/down-regulation, while βARKct gene therapy and subsequent GRK2 inhibition promote angiogenesis in this model (44). These results suggest that GRK2 expression level may negatively regulate angiogenesis in ischemic limb models.

### GRK2 as a Target for Anti-Angiogenesis Therapy

The above results suggest that regulating the GRK2-involved pathway in pathophysiological contexts characterized by abnormal angiogenesis leads to promising anti-angiogenesis therapy. Paeoniflorin-6’-O-benzene sulfonate (CP-25), a chemical modification derivative of paeoniflorin, has outstanding anti-inflammatory and soft regulation of inflammatory immune response (SRIIR) activities (77–80). As CP-25 can attenuate pannus formation in CIA and adjuvant arthritis (AA) models (81, 82), further studies have confirmed that the mechanism is closely related to GRK2. CP-25 inhibits PGE2-induced angiogenesis by downregulating EP4-AC-cAMP-PKA-mediated GRK2 translocation to the cell membrane, in parallel with reducing the inhibition of GRK2 by ERK1/2 (45). Furthermore, CP−25 inhibited pannus formation in the synovium of rats with AA, which may be associated with the downregulation of CXCL12/CXCR4 expression in the synovium (46). These findings provide a potential new mechanism for anti-angiogenic therapy with CP-25 in RA. Moreover, βARKct gene therapy in a hindlimb ischemia (HI) model induced GRK2 inhibition and promoted subsequent angiogenesis by suppressing ischemia-induced β_2_AR downregulation, but the detailed mechanism remains to be explored (44). Although these results suggest that GRK2 may be an effective target for antiangiogenic therapy in special disease models, whether this approach is still effective in tumors remains unknown.

Manipulation of tumor-associated angiogenesis represents a promising strategy to limit cancer progression; however, most of clinical tumor anti-angiogenesis drugs can easily cause drug resistance in patients (83–85). The main reason for drug resistance is that these drugs completely inhibit angiogenesis, which leads to an imbalance state that induces more severe angiogenesis. The results mentioned in this review suggest that either the low expression level of GRK2 or the excessive translocation activity of GRK2 can cause pathological angiogenesis. This may indicate that the imbalance and abnormal state of GRK2 expression and activity are the causes of pathological angiogenesis. Therefore, it is important to restore GRK2 to its normal state *via* soft regulation.

In this regard, we hope that GRK2 can be realized in the future as a new potential anti-angiogenesis therapeutic target in tumor therapy for tumor vascular normalization, which means a decrease in the number of vessels and an increase in perfusion (66, 86, 87). The tumor microenvironment is a complex network of multiple chemokines, metabolic reactions, and signaling cascades (88, 89). This requires in-depth exploration of the complex mechanism of GRK2 and various molecules in the tumor microenvironment in different tumor types in the future. The pathogenesis of different diseases is distinct and complex. Thus, it is important to study the relationship between GRK2 expression levels in specific cell types or in specific intracellular locations and angiogenesis for specific disease types.

## Conclusions And Perspectives

It has become clear that GRKs are multifunctional proteins that can interact not only with GPCRs but also with intracellular multiple non-GPCR proteins. The findings summarized in this review strongly suggest that changes in GRK2 expression levels and localization in ECs induced by distinct stimuli, may modulate GPCRs- and/or non-GPCRs-involved signaling through a variety of mechanisms, and further influence on cell proliferation, migration, and other behaviors. Moreover, these changes related to GRK2 may eventually lead to pathological angiogenesis in organisms. Under different disease conditions (in tumors or in RA), the GRK2 activity leading to EC activation and angiogenesis show opposite trends. Decreased GRK2 expression or the increased GRK2 translocation can both improve the activity of ECs, thus, promoting angiogenesis and disease progression. This suggests that the imbalance and abnormal activity of GRK2 may be the main cause of pathological angiogenesis. Therefore, when GRK2 is used as the target of anti-angiogenesis therapy, we should aim to restore the normal state of GRK2 activity, rather than completely inhibit or promote it.

However, there are still several issues remain to be resolved in the future. An increasing number of studies have shown that GRK2 expression levels, localization, and functional status are closely related to inflammation and inflammation-related diseases (3, 33, 90, 91). The network of inflammatory microenvironment components is complex. A better understanding of GRK2 specificity of expression and translocation mode in specific inflammation/pathological conditions will be pivotal to reveal the roles of this kinase in progression and other processes related to inflammation and diseases. Moreover, in view of existing research results, GRK2 may be a potential anti-angiogenesis therapy target in a variety of pathological conditions. However, there is a problem in that the activity of GRK2 affects angiogenesis in various ways in different diseases, including in different subtypes and different stages of progression of the same disease, such as in cancers (3, 42, 61). Thus, the specific contributions of GRK2 in angiogenesis and potential mechanisms therein, its integration in ECs and other cell types related to angiogenesis, and the expression and/or translocation changes that occur during disease development, need to be further confirmed. The above key issues should be addressed in future research to gain further insight into the roles of GRK2 and help in the design of GRK2-related therapeutic strategies for different diseases.

## Author Contributions

JK and CH conceived and designed this review. JK researched the literature and wrote the manuscript. WW is responsible for review, editing and funding acquisition. All authors contributed to the article and approved the submitted version.

## Funding

The study was supported with the Surface Project of National Natural Science Foundation of China (No. 81673444) and the Postdoctoral Science Foundation of Anhui Province (No. 2020B430).

## Conflict of Interest

The authors declare that the research was conducted in the absence of any commercial or financial relationships that could be construed as a potential conflict of interest.
